# Patterns and predictors of self-medication behavior of weight loss medications: a cross-sectional analysis of social media influence and role of pharmacist intervention

**DOI:** 10.3389/fphar.2025.1606566

**Published:** 2025-07-14

**Authors:** Neven Sarhan, Mona F. Schaalan, Azza A. K. El-Sheikh

**Affiliations:** ^1^ Clinical Pharmacy Department, Faculty of Pharmacy, Misr International University, Cairo, Egypt; ^2^ Biochemistry Department, Faculty of Pharmacy, Misr International University, Cairo, Egypt; ^3^ Basic Health Sciences Department, College of Medicine, Princess Nourah bint Abdulrahman University, Riyadh, Saudi Arabia

**Keywords:** weight loss medications, obesity, pharmacist, self medication users, social media influence

## Abstract

**Background:**

Misuse of weight-loss medicines, particularly those obtained via the internet and social media, raises critical concerns regarding patient safety, compliance, and risk of adverse drug reactions (ADRs).

**Objective:**

To evaluate patterns of weight-loss medication acquisition, the influence of social media on drug use behaviors, the role of pharmacist recommendations, and the key predictors of self-medication and adherence.

**Methods:**

A cross-sectional survey was utilized to assess sources of procurement of weight loss drugs, impact of social media on drug use, and sufficiency of pharmacist counseling on safety and drug compliance.

**Results:**

The study revealed that 53% of participants reported using weight-loss medications without consulting a physician. Among them, 32% acquired the medications directly from a pharmacy without prescription and 15% online through social media platforms. A significant majority (68%) indicated being strongly influenced by social media, with Instagram (45%) and TikTok (30%) being the most cited platforms. Pharmacist consultation was associated with improved adherence (50% vs. 25%, *p* = 0.0001) and enhanced awareness of drug safety (55% vs. 30%, *p* = 0.0004). Logistic regression revealed that self-medication was significantly predicted by younger age (18–30 years, OR = 2.1, 95% CI: 1.3–3.4, *p* = 0.002), frequent social media use (OR = 1.8, 95% CI: 1.1–2.9, *p* = 0.01), limited access to healthcare services (OR = 2.5, 95% CI: 1.4–4.4, *p* = 0.001), and prior unsuccessful weight loss attempts (OR = 1.9, 95% CI: 1.2–3.1, *p* = 0.005). In contrast, medication adherence was positively associated with pharmacist recommendations (OR = 2.34, 95% CI: 1.45–3.76, p-value <0.001), younger age (OR = 1.8, 95% CI: 1.1–2.9, *p* = 0.004), better access to healthcare (OR = 2.8, 95% CI: 1.6–4.7, p-value <0.001), low social media dependence (OR = 2.2, 95% CI: 1.3–3.6, *p* = 0.002) and previous medication adherence experience (OR = 1.7, 95% CI: 1.1–2.8, *p* = 0.012).

**Conclusion:**

Findings highlight a growing trend of social media-driven decisions regarding weight-loss medication use and the associated risks of unregulated online-purchases. Pharmacists play a crucial role in mitigating adverse outcomes by promoting drug safety and adherence. Policy efforts should focus on enhancing regulations of over-the-counter online sales, strengthening pharmacist-led patient education, and combating misinformation through evidence-based public health communication.

## Introduction

Obesity is a chronic and multifactorial condition with detrimental health-implications, including cardiovascular disease and various forms of cancer. Globally, an estimated 2.5 billion individuals are classified as overweight or obese—a figure projected to rise in the coming years (WHO, 2024a). Whilst lifestyle modification remain the first-line intervention for weight management, sustaining long-term weight loss through behavioral change alone is often challenging. This has led to a growing reliance on pharmacological treatments as adjuncts to lifestyle interventions (Jensen et al., 2014; Yumuk et al., 2015; WHO, 2024b).

Anti-obesity medications can support weight management by regulating appetite, metabolic rate, and nutrient utilization, with predominant classes being glucagon-like peptide-1 (GLP-1) receptor agonists, lipase-inhibitors, and CNS-stimulants, and other agents such as bupropion-naltrexone and topiramate (Gudbergsen et al., 2021; Larsen et al., 2017; Yanovski and Yanovski, 2014; Yanovski and Yanovski, 2021). While being effective when taken as directed by a physician, their misuse-including self-medication, off-label consumption, and illicit online purchases-has become a growing concern (Carneiro et al., 2017; Basch et al., 2025; Fittler et al., 2018).

The growing and threatening influence of social media and unregulated online health platforms has significantly shaped public perceptions and behaviors regarding the over-the counter use of weight loss drugs. Many individuals seek health advice from social media influencers, online forums, and non-verified websites, often bypassing healthcare professionals entirely (Basch et al., 2023; Fox et al., 2005; Xing et al., 2017). As a result, a considerable number of people obtain weight-loss drugs through unofficial channels, thus, missing necessary medical supervision (Chiappini et al., 2023). This can lead to increasing the risk of unsupervised use, overdose, exposure to counterfeit products, and severe side effects such as cardiovascular complications, metabolic disturbances, and gastrointestinal issues (Fong et al., 2024; Hendricks, 2017; Vrancken, 2015).

Self-medication in this context is particularly dangerous, as it often involves inadequate knowledge of appropriate dosing, potential drug interactions, and contraindications. Evidence suggests a growing trend in the unsupervised use of GLP-1 receptor agonists, largely fueled by online promotion and misinformation (Chiappini et al., 2023). Additionally, online purchasers of drugs are more vulnerable to being sold bogus and substandard products, which poses greater jeopardy to their health (FDA, 2024; WHO, 2024a).

Despite these mounting concerns, there is a notable lack of research exploring the prevalence, motivations, and consequences of self-medication behavior of weight-loss drugs. Understanding patient attitudes towards weight-loss medications, particularly their acquisition sources, awareness of associated risks, and their reliance on digital health information, is crucial for shaping effective public health strategies and regulatory policies (Gleason et al., 2024; Rodriguez et al., 2025).

Pharmacists are uniquely positioned to ensure optimizing weight control pharmacotherapy with efficient and rational management of weight-loss drugs. Their expertise in medication management, drug interaction, dosage adjustment, drug interaction screening, and patient education is instrumental in minimizing adverse drug reactions and enhancing treatment adherence (Aldaajani et al., 2024). Additionally, pharmacists contribute to pharmacovigilance efforts by monitoring patient-reported outcomes and identifying safety concerns linked to self-medication practices (Clements et al., 2021). They also serve a key role in counteracting misinformation by providing evidence-based guidance and correcting false claims found on social media and unverified health platforms (Correia et al., 2022).

This study aims to evaluate patterns of weight-loss medication acquisition, the influence of social media on drug use behaviors, the impact of pharmacist counseling on patient outcomes, and to identify key predictors of self-medication and adherence. The findings are intended to support evidence-based strategies that promote safer medication practices and improve public awareness around the risks of unregulated drug use.

## Materials and methods

### Study design

A cross-sectional study, employing a questionnaire to determine the prevalence, determinants of the self-medication behavior and adherence of slimming drugs (Supplementary Figure S1). This study was ethically approved by Misr International University Research Ethics Committee (IRB: MIU-IRB-2425-037) and conducted on strict compliance with ethical guidelines. The participants provided electronic consent, which guaranteed voluntary and informed participation in the study. Personal details were not requested, and the answers were anonymized. The survey was hosted on Google Forms, which allowed responses only from unique email accounts to ensure one submission per participant. Data were encrypted, and participant anonymity was preserved. Data were collected over a 4-week period from 15 February 2025, to 15 March 2025.

The questionnaire used in this study provided comprehensive data about the participants’ demographics, prescription drug use for weight loss, means of getting drugs, adherence to medical advice, subjective success with treatment, and utilizing health information from social media. Moreover, this study also analyzed the activities of pharmacists when attending to patients using drugs for weight loss, such as pharmacist intervention, counseling, and patient safety interventions, as well as pharmacovigilance reporting.

### Study population and sampling strategy

The inclusion criteria of this study involved participants who were 18 years or above and had used or intended to use weight loss drugs, with or without medical advice. Convenience sampling via social media and online forums was used to obtain a broad sample. In order to assess the role of pharmacists, the respondents were asked if they had received any counseling from a pharmacist. Inclusion criteria also involved Egyptian English and Arabic speakers with a history of current or past use of anti-obesity drugs, while exclusions included those below the age of 18, mentally disabled individuals, non-Egyptians, and physicians who prescribe weight loss drugs.

### Survey instrument development and structure

The questionnaire instrument was an English online self-report questionnaire with Arabic translation to further make it accessible and more understandable to a wider population. The questionnaire contained five big sections to obtain complete information regarding the use of weight loss medications and associated behaviors.

A. Demographic and health information: this section collected information on the participant’s age, gender, height, and weight to compute body mass index (BMI) and his/her known medical illness of obesity as well as medical history.
B. Treatment history and medication adherence: adherence was self-reported by participants using a series of structured questions related to missed doses, timing of intake, and intentional or unintentional discontinuation. These questions were adapted from validated adherence assessment models such as the Morisky Medication Adherence Scale (MMAS), though simplified for a general population.
C. The acquisition and use of weight-loss medication: this scale measured participants’ history of weight-loss medication use including the specific drugs taken (semaglutide, liraglutide, orlistat, phentermine, or more recently, tirzepatide). Participants reported their sources of acquisition—such as such as through a physician’s prescription, over-the-counter purchase via the internet, direct purchase from a pharmacy without a prescription, or recommendation from family or friends. The measure also captured the frequency and quantity of medication use. Additionally, participants indicated whether they consulted a healthcare professional prior to initiating treatment and whether they adhered to a medically prescribed self-care plan or engaged in unsupervised use of the medication.
D. Measurement of perceived effectiveness and attitudes and knowledge along with social media impact: by observing appetite suppression, weight loss outcome, and treatment satisfaction, trial participants assessed their weight loss medication’s effectiveness. Except for one open-ended question regarding the successful maintenance of weight upon drug withdrawal, participant satisfaction was rated on a Likert scale from 1 to 5 (1 Very Dissatisfied, 5 Very Satisfied). Moreover, participants’ knowledge concerning risks of use of weight-loss products such as side effects, contraindications, and regulators. Respondents were asked to agree or disagree with statements and report behaviors reflecting their beliefs and knowledge. The survey assessed claims related to safety verification methods, the influence and perceived reliability of social media, concerns about unregulated medication sources, beliefs about the pharmacist’s role in ensuring safety, and awareness of possible side effects and their information sources.
E. Role of pharmacists and patient safety: this section summarizes information on the role of pharmacists in ensuring patient safety. It assessed the role of the pharmacist in counseling for safe medication use and patient adherence. The section also examined knowledge and involvement in reporting adverse drug reactions (ADR) and pharmacovigilance activities. It also assessed the effect of advice from a pharmacist compared to health information obtained from the internet. Verifying the safety of weight-loss medications refered to actions such as reading the patient information leaflet, consulting healthcare professionals, or checking for regulatory approval and certifications.

The questionnaire was pilot-tested with a sample of 20 respondents to evaluate its clarity, relevance, and internal consistency. Participant feedback was used to refine and revise ambiguous or imprecise items to improve overall comprehension and content validity. The internal reliability of the scale was assessed using Cronbach’s alpha, yielding a coefficient of 0.84, which indicates a high level of internal consistency.

The questionnaire took 20–30 min, and data collection took place within 4 weeks to ensure a representative sample. Clinical significance was underscored by drug counseling, patient education, and ADR surveillance by pharmacists in keeping with international GPP standards (Chertes and Crisan, 2019).

### Sample size calculation

A sample size of 350, was calculated using standard formula for cross-section with 95% confidence, 50% estimated prevalence and 5% margin of error to provide sufficient power for statistical analysis in the event of anticipated non-responses.

### Statistical analysis

The survey data gathered was exported to Microsoft Excel to begin initial data cleaning and processing procedures. SPSS Statistics software (Version 22) was employed to analyze research data to identify patterns and relationships among variables. Categorical variables were presented as frequencies and percentages, whereas continuous variables were calculated as standard deviations and means. Categorical variable relationships were contrasted using the Chi-square test, and independent samples t-tests for continuous variables. Univariate analysis variables with p < 0.10 were entered into multivariate logistic regression models by stepwise backward elimination to identify independent predictors of self-medication behavior and medication adherence. Statistical significance was defined as p < 0.05 (two-sided). Odds ratios (ORs) and 95% Confidence Intervals (CIs) were calculated for all logistic regression results.

A sensitivity analysis was conducted excluding the 15 subjects (2.8%) who identified themselves as pharmacists to adjust for professional bias.

## Results

Demographic analysis of the study sample revealed that the average age of participants was 35.8 years. There was no significant difference in age between those who received pharmacist counseling (36.1 ± 9.8 years) and those who did not (35.5 ± 10.5 years) (p = 0.512). Gender distribution was balanced, with 45.6% of participants being male, and no significant difference observed between the two groups (p = 0.612). Both groups were also comparable in terms of Body Mass Index (BMI) categories (25–29.9 and >30 kg/m^2^) and educational status (high school, university degree, and postgraduate degree). The distribution of weight loss medications was similarly balanced between the two groups, including Semaglutide, Liraglutide, Orlistat, Phentermine, Tirzepatide, and the use of multiple medications. The prevalence of comorbidities—diabetes (18.6%), hypertension (22.1%), hyperlipidemia (15.3%), and thyroid disease (10.5%)—was also evenly distributed across both groups, indicating comparable baseline characteristics (p > 0.05), as detailed in Table 1.

**TABLE 1:** Demographic charactristics of participants.

Variable	Total Cohort (n = 542)	Received Pharmacist Counseling (n = 280)	Did Not Receive Pharmacist Counseling (n = 262)	p-value
Age (mean ± SD)	35.8 ± 10.2	36.1 ± 9.8	35.5 ± 10.5	0.512
Gender; n (%male)	247 (45.6%)	46.8%	44.2%	0.612
Body mass Index (BMI) (Kg/m^2^) (Mean ± SD)	28.3 ± 5.1	29.7 ± 4.3	28.8 ± 3.8	0.428
Overweight (BMI 25–29.9 Kg/m^2^)	244 (45%)	46%	43%	0.371
Obese (BMI ≥30 Kg/m^2^)	136 (25%)	26%	23.5%	0.561
Education Status - High school - University degree - Post-graduate degree	98 (18%) 356 (65.7%) 88 (16.3%0	49 (17.5%) 186 (66.4%) 45 (16.1%)	42 (16.1%) 179 (68.3%) 41 (15.6%)	0.702 0.683 0.396
Weight loss medications - Semaglutide - Liraglutide - Orlistat - Phentermine - Tirzepatide - Multiple medications * - Other	119 (22%) 70 (12.9%) 57 (10.6%) 47 (8.5%) 22 (4%) 101 (18.6%) 127 (23.4%)	78 (27.8%) 40 (14.2%) 26 (9.2%) 19 (6.8%) 12 (4.3%) 53 (18.9%) 52 (18.8%)	41 (15.6%) 30 (11.5%) 31 (11.8%) 28 (10.7%) 10 (3.8%) 48 (18.3%) 73 (28.3%)	0.721 0.872 0.284 0.182 0.472 0.501 0.121
Co-Morbidities (%) - Diabetes - Hypertension - Hyperlipidemia - Thyroid Disorder - Other	101 (18.6%) 119 (22%) 83 (15.3%) 57 (10.5%) 182 (35.5%)	54 (19.3%) 67 (23.9%) 45 (16.1%) 27 (9.8%) 87 (31%)	47 (17.8%) 52 (20.2%) 38 (14.4%) 30 (11.3%) 95 (36.3%)	0.682 0.418 0.756 0.619 0.528

Statistical test used: Independent t-Test applied to continuous variables and Chi-square Test for categorical variables; Significance levels at p < 0.05. * Multiple medications include combinations such as semaglutide + orlistat, liraglutide + phentermine, and tirzepatide + orlistat. Percentages may exceed 100% due to multiple responses.

The proportions of people who have verified the safety of weight loss medications compared to those who did not verify the safety are illustrated on Table 2. Four sources of medication are being contrasted: online retailers (e.g., Amazon, eBay), social media sites (e.g., Instagram, TikTok), unregulated direct sellers, and pharmacies/healthcare professionals (Table 2). Outcomes are such that pharmacies/healthcare providers have the highest proportion of verified-safe drugs (95.0%), while unregulated channels like social media and online marketplaces are significantly lower in their verifying rates (Table 2).

**TABLE 2:** Medication sources and safety awareness.

Medication Source	Total Cohort (n = 542)	Verified Safety (%)	Did Not Verify safety (%)	p-value
Online Marketplace (Amazon, eBay, etc.)	122 (22.5%)	37.5%	62.5%	0.01*
Social media (Instagram, TikTok, etc.)	109 (20.1%)	29.1%	70.9%	0.03*
Direct from Seller (Unregulated)	31 (5.7%)	33.6%	66.4%	0.02*
Pharmacy/Healthcare Provider	280 (51.7%)	95.0%	5.0%	0.0001*

Statistical test used: Chi-Square Test applied to categorical variables; Significance Levels at p < 0.05.

**FIGURE 1:**
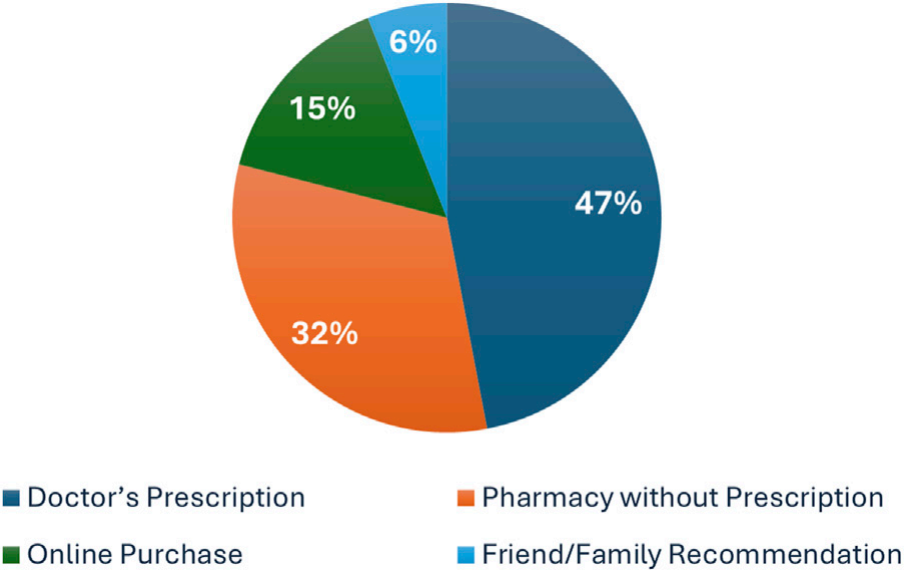
Pie Chart showing sources of weight loss medication purchases.

When asked about the source of their prescriptions, participants demonstrated a wide range of behaviors. Approximately 47% (n = 255) of those using weight loss medications reported obtaining them through a doctor’s prescription, while 32% (n = 173) purchased them without a prescription directly from a pharmacy. Additionally, 15% (n = 81) acquired their medications online—via social media or online stores—and 6% (n = 33) reported using drugs based on recommendations from friends or family members, as illustrated in Figure 1 and detailed in Supplementary Table S1. Overall, these findings indicate that nearly 53% of users accessed weight loss medications without medical consultation.

To assess the role of social media in influencing the use of weight loss medications, respondents who had used such drugs (n = 542) were asked about their sources of influence. Of these, 68% (n = 369) reported that social media played a role in their decision to use weight loss drugs. Of the total respondants, 45% (n = 244) cited Instagram as their primary source of information, followed by TikTok (30%, n = 163), Facebook (15%, n = 81), and YouTube (10%, n = 54), as detailed in Supplementary Table S2.

Analysis of adverse drug reactions (ADRs) demonstrated a significant protective effect of pharmacist counseling, with side effects reported by only 40.4% (n = 113) of participants who received counseling, compared to 62.2% (n = 163) among those who did not (p < 0.001). This significant reduction highlights the role of pharmacist intervention in preventing drug-related complications. Furthermore, a greater proportion of non-counseled patients (62.2%) experienced ADRs, compared to only 40.4% in the counseled group—further supporting the preventive impact of pharmacist-provided medication counseling (Table 3). The reported side effects were significantly higher in participants who did not receive counseling including the incidence of nausea, gastrointestinal distress, fatigue, ad mood changes.

**TABLE 3:** Total side effects experienced by participants.

Adverse Effect	Total Cohort (n = 542)	Received Pharmacist Counseling (n = 280)	Did Not Receive Pharmacist Counseling (n = 262)	Chi-Square (X^2^)	P-Values
Any Adverse Effect	298 (50.9%)	40.4% (113)	62.2% (163)	15.72	<0.001*
Nausea	190 (35%)	75 (26.8%)	115 (43.9%)	14.32	0.003*
Gastrointestinal Distress	163 (30%)	60 (21.4%)	103 (39.3%)	16.85	<0.001*
Dizziness	136 (25%)	50 (17.9%)	86 (32.8%)	14.01	0.017*
Fatigue	108 (20%)	45 (16.1%)	63 (24.0%)	5.36	0.021*
Mood Changes (Anxiety/Depression)	81 (15%)	32 (11.4%)	49 (18.7%)	5.72	0.017*

Statistical test used: Chi-Square Test applied to categorical variables; Significance level at p < 0.05. Percentages may exceed 100% due to multiple responses.

Pharmacist counseling had a positive impact on various patient outcomes (Supplementary Table S3). Awareness of the Pharmacist’s role was significantly higher among the counseled group (75%) compared to the non-counseled group (50%) (p = 0.0001), highlighting the importance of pharmacists in patient education. Additionally, medication safety screening was more frequently conducted in the counseled group (55% vs. 30%, p = 0.0004), further emphasizing the pharmacist’s contribution to ensuring medication safety. Additionally, the percentage of participants who experienced side effects was notably lower in the counseled group (35% vs. 55%, p = 0.0027), underscoring the prophylactic role of pharmacists in preventing adverse drug reactions. Adherence to medications was significantly higher among counseled patients (50% vs. 25%, p = 0.0001), further demonstrating the positive impact of pharmacist intervention on patient compliance, as shown in Table 4.

**TABLE 4:** Association of participants’ awareness of variable factors with pharmacy services.

Category	Received Pharmacist Counseling (n = 280)	Not Received Pharmacist Counseling (n = 262)	Chi-Square (X^2^)	p-value
Awareness of the role of Pharmacists (%)	75%	50%	16.1	0.0001*
Awareness of medication safety verification (%)	55%	30%	12.5	0.0004*
Incidence of potential side effects (%)	35%	55%	9.4	0.0027*
Awareness of social media influence (%)	40%	30%	6.2	0.014*
Awareness of the importance of medication adherence (%)	50%	25%	15.6	0.0001*
Concern about unregulated obesity medications (%)	60%	35%	10.3	0.0016*

Statistical test used: Chi-Square Test applied to categorical variables; Significance levels at p < 0.05. Percentages may exceed 100% due to multiple responses.

Logistic regression analysis identified several significant predictors of both self-medication and adherence to medication. For self-medication, individuals aged 18–30 years were more than twice as likely to self-medicate compared to older adults (OR = 2.1, p = 0.002). Excessive use of social media (OR = 1.8, p = 0.01), poor access to healthcare services (OR = 2.5, p = 0.001), and prior failed weight loss attempts (OR = 1.9, p = 0.005) were also associated with increased likelihood of using non-prescribed medications (Table 5).

**TABLE 5:** Logistic Regression Analysis Predicting Self-Medication Behavior and medication Adherence.

Predictor Variable	Odds Ratio (OR)	95% Confidence Interval	p-value
Self-medication Behavior			
Age (18–30 years vs. >30 years)	2.1	1.3–3.4	0.002
High Social Media Exposure	1.8	1.1–2.9	0.01
Lack of Regular Access to Healthcare	2.5	1.4–4.4	0.001
Prior Unsuccessful Weight Loss Attempts	1.9	1.2–3.1	0.005
Medication adherence			
Pharmacist Counseling	2.34	1.45–3.76	0.001
Age (18–30 years vs. >30 years)	1.8	1.1–2.9	0.004
Regular Access to Healthcare	2.8	1.6–4.7	0.003
Low Social Media Dependence	2.2	1.3–3.6	0.002
Previous Medication Adherence Experience	1.7	1.1–2.8	0.012

In terms of medication adherence, pharmacist counseling significantly improved compliance (OR = 2.34, p < 0.001), as did regular access to healthcare (OR = 2.8, p < 0.001) and low dependence on social media for medical decisions (OR = 2.2, p = 0.002). Medication adherence was higher among younger patients (OR = 1.8, p = 0.004), while those with a previous history of adherence were more likely to remain compliant (OR = 1.7, p = 0.012), as shown in Table 5. Of the 542 people questioned, 15 (2.8%) identified themselves as being pharmacists. A sensitivity analysis limiting the data to these individuals was conducted for assessment of potential professional bias. Results of logistic regression were not significantly altered, confirming the stability of found associations.

## Discussion

The results of this study provide concrete evidence for the critical role of pharmacy services in improving drug adherence, enhancing safety, and reducing adverse drug reactions (ADRs). The study also explored the prevalence, causes, and consequences of self-medication behavior of weight loss medications, particularly focusing on self-medication, e-commerce drug acquisition, and the influence of social media on drug-taking behavior. Findings revealed that the majority of participants used weight-loss drugs without a prescription, relying on non-clinical sources such as internet support groups, illicit online pharmacies, and informal recommendations from family or friends.

These results align with the findings of Chiappini et al. (2023), who used data from the FAERS database and reported a growing trend of off-label and unsupervised use of semaglutide for weight loss (Chiappini et al., 2023). According to their study, around 6% of semaglutide users were prescribed the medication without medical oversight, a trend similarly observed in our sample. In line with existing literature, this study confirms the presence of GLP-1 receptor agonists in unregulated online supplements and off-label drugs sold without proper control (Gleason et al., 2024; Rodriguez et al., 2025; Cameron et al., 2022; Marcum et al., 2021).

Our results further revealed the significant role social media plays in shaping participants’ decisions regarding weight-loss medications. Respondents frequently referred to advertisements, peer-to-peer, word-of-mouth communication, social media posts, and non-verified health websites. These observations are consistent with the findings of Huang et al. (2020) and others (Carneiro et al., 2017; Basch et al., 2023; Fittler et al., 2018), who emphasized the impact of social media on promoting self-medication, especially among individuals seeking rapid weight loss. Their analysis of online marketing strategies for weight-loss products showed a pattern of exaggerated benefits and downplayed risks, contributing to increased consumer demand and unsafe medication practices.

This trend was reinforced by our study’s findings, which show that a substantial proportion of participants bypassed standard medical consultations and opted for information from electronic marketplaces or peer sources. This aligns with Yanovski and Yanovski (2014), who stressed the need for close monitoring of obesity drug regimens due to the risks associated with abrupt cessation, including rebound weight gain, psychological distress, and heightened cravings (Yanovski and Yanovski, 2014).

Our study also documented a clear disparity between individuals who self-medicated and those who received pharmacist counseling. While previous studies have demonstrated the positive effects of pharmacist intervention on medication adherence and adverse outcomes (Aldaajani et al., 2024; Clements et al., 2021; Adisa et al., 2024), our results reinforce this by showing improved safety and reduced potential for misuse among counseled patients (Chandak, 2023; Kovačević et al., 2024). The most commonly reported side effects included gastrointestinal discomfort, dizziness, fatigue, and mood changes—findings that are consistent with prior pharmacovigilance studies on GLP-1 receptor agonists (Fong et al., 2024; Hendricks, 2017; Vrancken, 2015).

Importantly, ADRs were significantly lower among patients who received pharmacist counseling (40.4% vs. 62.2%, p < 0.001), emphasizing the preventive role of pharmacists in identifying drug-related risks and guiding appropriate medication use. These findings are supported by a systematic review by Zavaleta-Monestel et al. (2024), which demonstrated similar reductions in ADRs among patients enrolled in pharmacist-led medication therapy management programs (Zavaleta-Monestel et al., 2024). Pharmacy-led interventions such as medication therapy management and patient education have been observed to increase medication safety and avoid complications by proper utilization and reduction of treatment anxiety (Naseralallah et al., 2024b; Umeh et al., 2025).

Medication adherence was also markedly higher among counseled patients (50% vs. 25%, p = 0.0001), underscoring the importance of patient–pharmacist interactions in enhancing compliance. This is consistent with literature documenting improved outcomes due to pharmacist interventions (Jacob et al., 2022)**.** Pharmacist counseling also had a strong influence on patients’ verification behavior, as seen in the significantly higher affirmation of drug safety among counseled patients (55% vs. 30%, p = 0.0004). This supports the conclusion that pharmacist guidance increases patient vigilance, reducing the likelihood of self-medication and counterfeit drug use.

In contrast, patients purchasing drugs from online platforms or via social media showed lower rates of medication legitimacy affirmation (37.5% and 29.1%, respectively, p < 0.05), highlighting the need for pharmacist-led education and public awareness campaigns. These findings echo other research pointing to the dangers of unregulated online pharmacies and the health risks they pose (Rosalind et al, 2021).

The side effect frequency chart further supports the value of pharmacist intervention. Patients who were counseled reported fewer respondants with adverse effects compared to those who were not (35% vs. 55%, p = 0.0027). These findings suggest that pharmacists play a key role in optimizing treatment regimens and preventing drug–drug interactions (Naseralallah et al., 2024a).

One of the most concerning findings from this study is the widespread lack of awareness regarding the proper use of weight-loss medications. Many participants did not realize that drugs such as GLP-1 receptor agonists and appetite suppressants are intended for specific medical conditions, may be contraindicated in others, and carry significant risks if misused. This is in agreement with Jensen et al. (2014), who found that patients were often not informed about the long-term consequences of pharmacologic obesity treatments, resulting in improper use and heightened risk (Jensen et al., 2014; Yumuk et al., 2015).

Additionally, a majority of participants indicated greater trust in social media and internet forums over healthcare professionals when selecting weight-loss drugs. This trend is especially concerning, as it marks a departure from evidence-based medical practice and promotes exposure to potentially misleading or false health information. The WHO (2024a) obesity report emphasized the urgent need for improved patient education and healthcare provider engagement to close such knowledge gaps (WHO, 2024a).

Forest plot analyses identified, younger age, high social media exposure and lack of regular access to healthcare as significant predictors of self-medication. This underscores the influence of digital platforms in shaping medication behavior, and the risks posed by online misinformation. In contrast, better medication adherence was associated with frequent healthcare visits, pharmacist counseling and low social media medication dependence—findings supported by studies showing that formal pharmacist follow-ups improve long-term treatment adherence and health outcomes (Hovland et al., 2020).

Despite its insights, this study has several limitations. Primarily, the use of self-reported data introduces potential recall bias, as participants may inaccurately recall or report their medication behaviors. Additionally, the use of convenience sampling limits the generalizability of findings to the broader population of weight-loss medication users, possibly hampering the external validity of the study. Another important limitation of this study is the use of social media which may have introduced selection bias by overrepresenting digitally active individuals, potentially skewing results related to social media influence on weight-loss medication use. As a result, the findings related to social media influence should be interpreted with caution and may not fully reflect the experiences of less digitally engaged populations. Additionally, while physicians were excluded to minimize prescriber bias, pharmacists were included due to the study’s focus on their counseling role; however, their inclusion may have introduced a degree of professional bias that could influence responses related to pharmacist intervention.

Future research should include longitudinal designs to assess the long-term effects of self-medication, online drug purchases, and social media influence. Moreover, qualitative studies exploring psychological motivations for avoiding physician consultations, and in-depth analyses of influencer marketing and online advertising in the context of anti-obesity drugs, would enhance the development of targeted public health interventions.

## Conclusion

This study addresses growing concern regarding self-medication behavior of weight loss medication, especially self-medication, online purchase of medicines, and internet misinformation. The findings underscore the necessity for improved patient education, tighter regulatory oversight, and more engaged healthcare professional involvement to facilitate safe and responsible use of weight loss medication as well as out the immediate necessity for increased pharmacist involvement in the prescription of weight loss medications. Such studies will contribute to the evidence base for pharmacist-provided medication counseling and weight management and medication safety. To counteract the excess demand for pharmaceutical weight loss treatment, a multi-faceted response involving policy reform, public health intervention, and digital health practice is necessary in addition to pharmacist-delivered medication counseling.

## Data Availability

The raw data supporting the conclusions of this article will be made available by the authors, without undue reservation.
