# Supplementary material for: Patterns and predictors of self-medication behavior of weight loss medications: a cross-sectional analysis of social media influence and role of pharmacist intervention
Source: Front Pharmacol. 2025 Jul 14;16:1606566. doi: 10.3389/fphar.2025.1606566 (PMC12301901; doi:10.3389/fphar.2025.1606566)
Supplement: Supplementary file 1 [file Supplementaryfile1.docx]

Patterns and Predictors of Irrational Use of Weight Loss Medications: A Cross-Sectional Analysis of Social media influence and Role of Pharmacist Intervention

**Supplementary Material**

Neven Sarhan ^1^*, Mona F. Schaalan ^2^ and Azza A.K. El-Sheikh ^3^

^1^ Clinical Pharmacy Department, Faculty of Pharmacy, Misr International University, Cairo, Egypt; [nevine.mohamed@miuegypt.edu.eg](mailto:nevine.mohamed@miuegypt.edu.eg)

^2^ Biochemistry Department, Faculty of Pharmacy, Misr International University, Cairo, Egypt; [mona.schaalan@miuegypt.edu.eg](mailto:mona.schaalan@miuegypt.edu.eg)

^3^ Basic Health Sciences Department, College of Medicine, Princess Nourah bint Abdulrahman University, P.O. Box 84428, Riyadh 11671, Saudi Arabia; [aaelsheikh@pnu.edu.sa](mailto:aaelsheikh@pnu.edu.sa)

* Corresponding author: Neven Sarhan. Adress: Villa 201 street 44, New Cairo, Cairo, Egypt, 11315. Email: [nevine.mohamed@miuegypt.edu.eg](mailto:nevine.mohamed@miuegypt.edu.eg)

***** Correspondence: [nevine.mohamed@miuegypt.edu.eg](mailto:nevine.mohamed@miuegypt.edu.eg); Tel.: +201023999996

**Figure S1. Study flow chart**

**
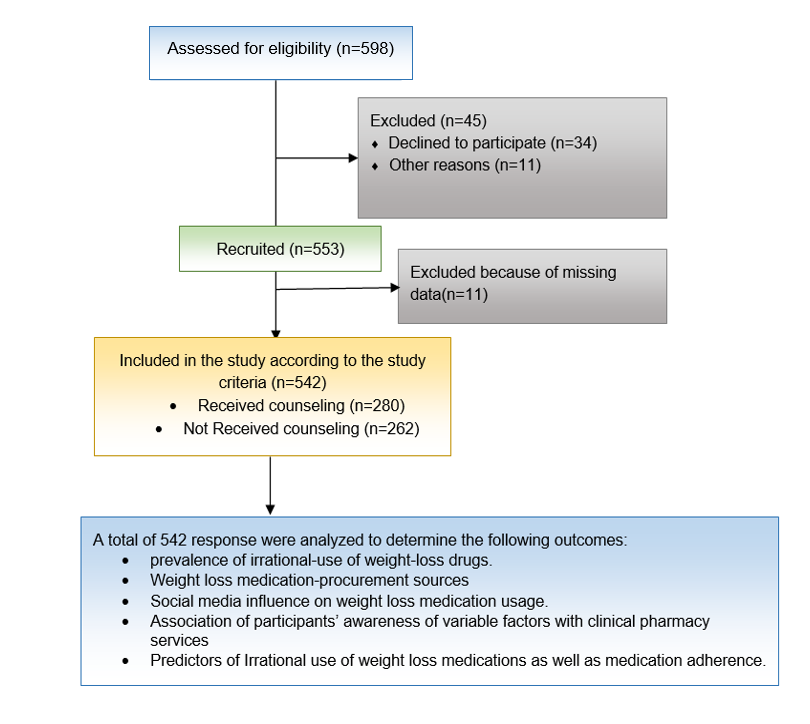
**

**Table S1. Sources of Medication Acquisition Among Medication Users**

| **Source of weight loss medication** | **Percentage (%)** | **Number**  **(n= 542)** |
| --- | --- | --- |
| Doctor’s Prescription | 47% | 255 |
| Pharmacy dispensing without Prescription | 32% | 173 |
| Online Purchase without Prescription | 15% | 81 |
| Friend/Family Recommendation | 6% | 33 |

**Table S2. Social Media Influence on Medication Use**

| **Social Media Influence** | **Percentage (%)** | **Number**  **(n= 542)** |
| --- | --- | --- |
| Reported Social Media Influence | 68% | 369 |
| Primary Platform – Instagram | 45% | 244 |
| Primary Platform – TikTok | 30% | 163 |
| Primary Platform – Facebook | 15% | 81 |
| Primary Platform – YouTube | 10% | 54 |
| Followed Health Influencers | 40% | 217 |

**Table S3. Role of Pharmacist in rational use of weight loss medications**

| **Pharmacist-Provided Information** | **Percentage (%)** | **Number**  **(n= 280)** |
| --- | --- | --- |
| Proper dosage and administration | 87% | 244 |
| Drug interactions | 68% | 190 |
| Side effects and risk awareness | 72% | 202 |
| Pharmacovigilance (ADR reporting) | 14% | 39 |
