# Supplementary material for: Patterns and predictors of self-medication behavior of weight loss medications: a cross-sectional analysis of social media influence and role of pharmacist intervention
Source: Front Pharmacol. 2025 Jul 14;16:1606566. doi: 10.3389/fphar.2025.1606566 (PMC12301901; doi:10.3389/fphar.2025.1606566)
Supplement: Supplementary file 2 [file Supplementaryfile2.docx]

**Survey about Patient Perspectives on Weight Loss Medications**

**Online Consent Form**

**Study Title**
**Assessment of the Irrational Use of Weight Loss Medications: A Cross-Sectional Study**
You are invited to participate in a research study. This study aims to assess the prevalence and factors contributing to the irrational use of weight loss medications. Your participation is entirely voluntary, and the survey will take approximately [20-30] minutes to complete.

**Purpose of the Study**

The objective of this study is to gather insights into the patterns of weight loss medication usage and understand factors associated with their irrational use.

**Participation Details**

- **Confidentiality**
  Your responses will remain anonymous and confidential. No personally identifiable information (PII) or personal health information (PHI) will be collected.
- **Voluntary Participation**
  Participation is entirely voluntary. You may withdraw at any time without penalty or providing a reason.
- **Survey Format**
  The survey consists of questions related to the use of weight loss medications. No sensitive or identifying questions will be asked.

**Potential Risks and Benefits**

- **Risks**
  There are no anticipated risks associated with participating in this study.
- **Benefits**
  While there are no direct benefits to you, your responses will contribute to a better understanding of medication use patterns, which could inform public health strategies.

**Data Security**

- Responses will be collected through a secure online platform.
- Data will be encrypted during transmission and securely stored.
- Only authorized researchers will have access to the anonymized data.

**Consent**

By clicking the "I Agree" button below, you indicate that:

1. You have read and understood the information provided.
2. You voluntarily agree to participate in this study.
3. You are at least 18 years of age.

[ ] **I Agree**
[ ] **I Do Not Agree**

**Section 1: Demographics and Health Background**

1. **What is your age? ***

- 18 to 24
- 25 to 34
- 35 to 44
- 45 to 54
- 55 to 64
- 65 or over

1. **What is your gender? ***

- Male
- Female

1. **What is your current height and weight? ***

**____________________________________**

1. **Have you been diagnosed with obesity or overweight by a doctor? ***

- Yes
- No

1. **Do you have any of the following health conditions? (Select all that apply) ***

- Type 2 Diabetes
- High blood pressure
- High cholesterol
- Sleep Apnea
- Joint pain or arthritis
- Other: ____________

1. **Are you currently receiving any medical treatment for other health conditions? ***

- Yes
- No

**Section 2: Treatment History & Medication Adherence**

1. **Are you currently using weight loss medication? ***

- Yes
- No

1. **If yes, which medication are you using? ***

- Mounjaro (Tirzepatide)
- Saxenda (Liraglutide)
- Ozempic
- Orlistat
- Other: ____________

1. **What was your reason for using the medication? ***

- To lose weight
- To manage type 2 diabetes
- Both weight loss and diabetes management
- Other: ____________

1. **How long have you been using this medication? ***

- Less than 3 months
- 3–6 months
- 6–12 months
- More than 1 year

1. **How effective was the medication for weight loss? ***

- Very effective
- Somewhat effective
- Not effective
- Unsure

1. **Are you following any lifestyle changes along with the medication? ***

- Yes, diet only
- Yes, exercise only
- Yes, both diet and exercise
- No

1. **Were you able to maintain the weight loss after stopping the medication? ***

- Yes
- No
- Still using the medication

1. **How satisfied are you with the results of the medication? ***

- Very satisfied
- Satisfied
- Neutral
- Dissatisfied
- Very dissatisfied

1. **How much weight did you lose while using the medication? ***

- 0–5% of body weight
- 5–10% of body weight
- 10–15% of body weight
- More than 15% of body weight
- I did not lose weight

1. **Do you believe it is important to follow a prescribed medication schedule? ***

- Yes
- No

1. **In the past week, how many times did you miss taking your weight-loss medication? ***

- 0 times
- 1–2 times
- 3–5 times
- More than 5 times
- I don’t remember

1. **Do you sometimes forget to take your medication? ***

- Yes
- No

1. **When you feel better, do you sometimes stop taking your medication? ***

- Yes
- No

1. **When you feel worse after taking your medication, do you stop taking it? ***

- Yes
- No

1. **Have you ever missed a dose because you were traveling or away from home? ***

- Yes
- No

1. **Have you skipped any doses due to the medication being too expensive? ***

- Yes
- No

1. **What are the main reasons you have missed doses? (Select all that apply) ***

- I forgot
- I was busy
- I didn’t feel the medication was helping
- I experienced side effects
- Cost of medication
- I ran out of pills
- Other: ____________

1. **Have you ever stopped taking weight-loss medication without medical advice? ***

- Yes
- No

**Section 3: Side Effects ‘detection**

1. **Did you experience the following side effects? ***

- Nausea or vomiting
- Abdominal pain or discomfort
- Constipation or diarrhea
- Dizziness or lightheadedness
- Headaches
- Blood sugar changes
- Fatigue or tiredness
- Heartburn, reflux, or indigestion
- Appetite changes
- Skin reactions (rash, itching)
- Swelling (hands, feet, face)
- Irregular heartbeat or palpitations
- Mood changes (anxiety or depression)
- Hair loss or thinning
- Insomnia
- Vision changes
- Shortness of breath or chest discomfort
- Excessive thirst or dehydration
- Symptoms of paralytic ileus

1. **Are there any other symptoms you experienced that were not mentioned above? ***

- Yes: ____________
- No

**Section 4: Source, Safety, and Influence Factors**

1. **Did you consult a healthcare provider before starting the medication? ***

- Yes
- No

1. **Who prescribed the medication to you? ***

- Doctor
- Pharmacist
- Self-prescribed
- Friend/Family recommendation
- Online (no prescription)

1. **Where did you purchase the medication? ***

- Online marketplace (Amazon, eBay)
- Social media (Instagram, TikTok)
- Direct from individual/seller
- Pharmacy or hospital
- Other: ____________

1. **Did you verify the safety of the medication before purchase? ***

- Yes, checked regulations/certifications
- No, assumed it was safe
- No, was unaware of risks

1. **How influential was social media in your decision to use the medication? ***

- Very influential
- Somewhat influential
- Not influential at all

1. **Have you ever purchased a medication after seeing it on social media? ***

- Yes
- No

1. **Do you believe social media information about medications is reliable? ***

- Strongly agree
- Agree
- Neutral
- Disagree
- Strongly disagree

1. **How concerned are you about the risks of unregulated medications? ***

- Very concerned
- Somewhat concerned
- Not concerned

1. **Would you use a weight-loss drug from a non-medical source? ***

- Yes
- No

**Section 5: Medication Counseling and Awareness**

1. **Did a pharmacist provide counseling about your weight-loss medication? ***

- Yes
- No

1. **Do you believe pharmacists play an important role in ensuring medication safety? ***

- Strongly agree
- Agree
- Neutral
- Disagree
- Strongly disagree

1. **Which steps did you take to verify your medication's safety? ***

- Read the patient leaflet
- Consulted pharmacist/physician
- Verified official website
- Did not verify
- Other: ____________

1. **Were you aware of possible side effects before taking the medication? ***

- Yes
- No

1. **Who informed you about the side effects? ***

- Doctor
- Pharmacist
- Internet
- Social media
- I did not know
- Other: ____________

**Thank you for your participation!**
